# Developmental Relations Between Peer Victimization, Emotional Symptoms, and Disability/Chronic Condition in Adolescence: Are Within- or Between-Person Factors Driving Development?

**DOI:** 10.1007/s10964-024-02114-3

**Published:** 2024-12-04

**Authors:** Russell Turner, Ylva Bjereld, Lilly Augustine

**Affiliations:** 1 Department of Social Work, University of Gothenburg, Gothenburg, Sweden; 2 School of Education and Communication, Jönköping University, Jönköping, Sweden

**Keywords:** Peer victimization, Bullying victimization, Emotional symptoms, Disability, Chronic condition

## Abstract

Although there is a link between peer victimization, emotional symptoms, and disability or chronic condition in adolescence, less is known about the role of stable, individual differences compared to developmental processes. The current study addressed this gap by examining developmental relations between peer victimization, emotional symptoms, and disability or chronic condition. Three waves of self-report panel data on Swedish adolescents at ages 13, 15, and 17 years (*n* = 734; 51.6% girls) were used. Data were analyzed using random-intercept cross-lagged panel models with invariance tests by sex, disability/chronic condition, and family affluence. Girls and adolescents with a disability or chronic condition showed more heterogeneity in the co-development of peer victimization and emotional symptoms, with girls being more prone than boys to developing emotional symptoms following peer victimization, and particularly in early adolescence. Girls and adolescents with a disability or chronic condition had elevated within-person levels of both peer victimization and emotional symptoms throughout adolescence. Theory and practice may need to pay additional attention to the role of sex, disability, and their intersection, as well as age, regarding the development of peer victimization and emotional symptoms.

## Introduction

Adolescents with a disability or chronic condition are more likely to be victimized by their peers (Pinquart, 2017; Sentenac et al., 2013). In general adolescent populations, being victimized is linked with several adverse psychological outcomes during adolescence, particularly internal or emotional symptoms such as anxiety or depression (Kwan et al., 2020; Polanin et al., 2021; Zwierynska et al., 2013). However, adolescents with a disability or chronic condition are likelier to have emotional symptoms, irrespective of peer victimization (Hatton et al., 2018), and pre-existing emotional symptoms are a risk factor for peer victimization (Fekkes et al., 2006; Park et al., 2020). Thus, adolescents with a disability or chronic condition may be not only subject to increased peer victimization due to their disability, but also experience elevated levels of emotional symptoms that may be due to victimization, their disability, or both. While many studies support a bi-directional relation between peer victimization and emotional symptoms in general adolescent populations (Christina et al., 2021), it is unknown whether these relations concern temporal, developmental processes during adolescence or stabler, trait-like individual differences, such as a disability or chronic condition. Further, there is little research on how a disability or chronic condition relates developmentally to peer victimization and emotional symptoms during adolescence. For example, does the link between disability or chronic condition and peer victimization or emotional symptoms change during adolescence? The current study aims to address these gaps with prospective, longitudinal data over three waves covering ages 13, 15, and 17 from over 700 Swedish school children.

### Developmental Relations between Peer Victimization and Emotional Symptoms

Peer victimization is often defined as repeated or continuous aggression conducted by one or more peers with the intent to hurt, harm, or gain control over another person (Olweus, 1993). An adolescent is thus considered victimized when he or she is repeatedly exposed to negative actions from one or more peers from which the victimized adolescent has trouble defending him/herself (Olweus, 1993). Peer victimization, by definition, is thus an enduring part of the victim’s developmental process. In general populations, it tends to peak in early adolescence, e.g., age 12-13, and decreases during middle and late adolescence (Biswas et al., 2020; Kennedy, 2021). However, once a victim, the likelihood of remaining a victim during adolescence is between 55 and 60 per cent (Zych et al., 2020). This suggests that for some adolescents, victimization does not abate in mid-adolescence. The development of emotional symptoms in adolescents generally follows an opposite pattern to peer victimization, rising towards mid-adolescence (Inchley et al., 2020) and peaking in late adolescence (Svensson et al., 2020). There is also evidence that early emotional symptoms are linked to continued emotional symptoms throughout adolescence (e.g., Ferdinand et al., 2007; Gregory et al., 2007).

Given that peer victimization and emotional symptoms during adolescence have similar reciprocal effects (with r-values of .2) (Christina et al., 2021), there is a question of why two opposite trends occur, rather than an escalating amplification effect of these temporal trends on each other during adolescence. An explanation is that temporal, dynamic changes play less of a role than trait-like individual differences, e.g. the decrease in victimization and increase in emotional symptoms are mainly driven by between-person factors (e.g. sex) that are present and prior to, and roughly stable during, adolescence. For example, boys tend to be more frequently victimized than girls (Smith et al., 2019) whereas girls tend to have higher levels of emotional symptoms than boys, especially in late adolescence (Patton et al., 2008). Of the studies that support bi-directional links between peer victimization and emotional symptoms (see Christina et al., 2021), none have analyzed whether the link is stronger at the between-person (trait-like) level or at the within-person (e.g. temporal change in emotional symptoms) level. Indeed, failing to separate between-person variance from temporal change means that previous studies may be conflating two different developmental processes, that is, trait-like development from the effect of temporal, dynamic events. For example, while Christina et al. (2021) state that neither age nor sex moderated the bi-directional links between peer victimization and emotional symptoms, this is based on a potential conflation of between- and within-person processes. It may be that individual-level moderators, such as sex, do matter but only concerning temporal, within-person processes. In general, failing to separate between- and within-person processes may over-estimate the strength of links between peer victimization and emotional symptoms (see Curran & Bauer, 2011; Keijser, 2015). Understanding whether stable, trait-like processes or temporal events better explain the relations between peer victimization and emotional symptoms would be an essential part of a theoretical account of the development of these factors in general adolescent populations.

### Relations between Disability or Chronic Condition and the Development of Peer Victimization and Emotional Symptoms

Defined using the International Classification of Functioning, Disability, and Health—Children and Youth version (WHO, 2001), a disability or chronic condition is something that limits participation in daily life activities. Such a functional impairment, if continuous during adolescence, may be viewed by peer victimizers as an outward social marker of potential weakness or difference, which may explain why adolescents with a disability or chronic condition have a higher risk of chronic and repeated victimization (Bjereld et al., 2022) and are twice as likely to be victimized as peers without a disability or chronic condition, even when controlling for confounding variables, such as family affluence and social support (Sentenac et al., 2013). Theoretically, the mechanisms driving the increased victimization of adolescents with a disability or chronic condition may lie in dominant ableist norms at a macro-level, e.g. that disability is generally viewed negatively in society (see Charlton, 2006), but also in moral justification and/or victim attribution at an individual level, e.g. victimizers ‘justifying’ their actions to themselves as an achievement and that the victim deserves the negative treatment (Thornberg & Jungert, 2014).

The link between peer victimization and emotional symptoms, seen in general adolescents, is made more complex when considering adolescents with a disability or chronic condition as they are also more likely to have emotional symptoms irrespective of peer victimization, compared to adolescents without a disability or chronic condition (Hatton et al., 2018). A cumulative adversity mechanism may be at work, in that adolescents with a disability have been shown to have higher exposure from an earlier age to adversities, such as poverty and parental mental health problems, than those without disabilities, and these adversities in turn increase the likelihood of poorer mental health more so for adolescents with a disability (Emerson & Hatton, 2007; Emerson et al., 2006). Adolescents with a disability from lower socio-economic backgrounds also tend to have elevated levels of peer victimization, compared to those from higher socio-economic backgrounds (Kavanagh et al., 2017). Thus, stable factors such as poverty/socio-economic class may have a moderating role on the links between peer victimization, emotional symptoms, and disability or chronic condition, though it is unclear whether poverty affects temporal, developmental (within-person) relations.

Given the stability over time of a disability or chronic condition, it may function as a key individual-level stable mechanism that exerts a continued influence on developmental processes in adolescence, particularly on peer victimization and emotional symptoms. However, little is known about whether temporal relations between peer victimization and emotional symptoms differ for adolescents with a disability or chronic condition. Understanding whether adolescents with a disability or chronic condition have different developmental processes to general adolescent populations would support tailoring of interventions, concerning both peer victimization and emotional symptoms, as well as being important for theoretical work.

## Present Study

Few studies analyze how a disability or chronic condition is linked longitudinally during adolescence to peer victimization and emotional symptoms, and there are none that compare within- and between-person processes for adolescents with or without a disability or chronic condition. The present study thus assesses the co-development of peer victimization and emotional symptoms with two main research questions. The first question concerns adolescents in general: i) do stable, individual (between-person) factors explain the developmental relations between peer victimization and emotional symptoms, more so than temporal (within-person) processes? ii) What is the strength of temporal, developmental relations between peer victimization and emotional symptoms. The hypotheses are: i) individual differences will play a greater role than temporal processes; ii) relations at the within-person level are expected to be weak, if at all present, and constant over the three time points reflecting a low, but continuous effect of temporal processes, and a stronger role of individual, stable factors. The second research question focuses on adolescents with a disability or chronic condition: i) are the developmental relations between peer victimization and emotional symptoms different for adolescents with a disability or chronic condition, and ii) if so, what are the links between having a disability or chronic condition and peer victimization and emotional symptoms at different developmental stages during adolescence? For both research questions, the moderating role of sex and family affluence as an indicator of socio-economic class is explored. For the second research question, the hypotheses are: i) developmental relations between peer victimization and emotional symptoms will be different for adolescents with a disability or chronic condition, with between-person factors playing a greater role than within-person factors, and playing a greater role for adolescents with a disability or chronic condition than those without a disability or chronic condition; ii) the developmental relations between disability or chronic condition and peer victimization and emotional symptoms will be similar throughout adolescence, reflecting the stability of the effect of having a disability or chronic condition. Additionally, both moderators – sex and family affluence – are expected to exert an influence in all models.

## Method

### Measures

#### Peer victimization

Data was gathered using a self-report questionnaire. Peer victimization was measured using four question items: 1) “Sometimes you can be rejected by one or more of your peers and not allowed to join in. Has this happened to you this term?” 2) “Have you been hit, kicked, or attacked by someone in school or on your way to or from school this term?” 3) “Have you been teased in an unpleasant way, or has someone said nasty things to you in school or on your way to and from school this term?” 4) “Has anyone shown or sent offensive pictures, photos, drawings, or messages to you this term?”. Response categories were 1 = Never, 2 = Yes sometimes, 3 = Yes, often. The first three questions were taken from the measure used by Özdemir and Stattin (2011). The fourth question was added to capture cyber-victimization (see Ybarra et al., 2012). In an international report using EU kids online data, approximately 10% of children aged 9 to 16 reported being a victim of online bullying in most of the countries (Smahel et al., 2020). Cyber-victimization may have more commonalities than differences with face-to-face victimization (Tokunga, 2010) and thus warrants the inclusion of an item on cyber-victimization. A confirmatory factor analysis (at t1) showed good fit (χ2 = 0.38, df = 2, *p* = 0.83; CFI = 1.00; RMSEA < 0.01; SRMR = 0.01), though due to the complexity of the modeling, a mean score was used, allowing one missing answer.

#### Emotional symptoms

Emotional symptoms were measured using Goodman et al.’s (1997) Strengths and Difficulties Questionnaire (SDQ) sub-scale. The scale comprises five items such as “I worry a lot” and “I am often unhappy, downhearted, or tearful”. Response categories were 0 = Not true, 1 = Somewhat true, 2 = Certainly true. A mean score, rather than a sum, was calculated, allowing one missing answer, to achieve comparability with the peer victimization measure. Cronbach’s alpha for the five items was 0.7.

#### Disability or chronic condition

The current study used a functional description of disability or chronic condition (D/CC) in line with the WHO (2001) classification. A dichotomous measure was created from a list of 18 pre-determined disability categories (including an “Other” option) in combination with the participant’s own assessment of the level of difficulty the disability creates in everyday life (using three response categories “Mild”, “Medium”, and “Difficult”). Functional impairment, rather than diagnosis, is thus used as the meaningful unit of analysis (see Kavanagh et al., 2017). The list of 18 categories included a range of disabilities such as diabetes, reduced sight, speech problems, epilepsy, asthma, ADHD, and general intellectual difficulty. Participants who answered “Yes” to at least one of the categories of disability and either “Medium” or “Difficult” to the level of functional difficulty were coded as having a D/CC (0 = No, 1 = Yes). The disability categories did not include any items that relate to emotional symptoms or similar mental health issues, thus maintaining a clear distinction between the concepts of functional impairment and emotional symptoms.

#### Family affluence

Family affluence was measured using the item: “How are your family’s finances compared to other families where you live?” with three response categories: 1 = Less money than other families, 2 = The same as other families, and 3 = More money than other families. A similar subjective measure has been used extensively in the Health Behaviors in School-Children (HBSC) studies in over 51 countries and numerous studies (see Ahlborg et al., 2017).

#### Sex

Sex was measured once using a self-report, dichotomous question item with two response categories: “Female” and “Male”.

### Participants and Internal Drop-Out

Three waves of data were used from the Longitudinal Research on Development in Adolescence (LoRDIA) research program, which was a prospective, total population study in four municipalities in west Sweden, comprising two panels of youth a year apart in age, sampled contemporaneously. All adolescents registered at all schools, including special schools (*särskolan*), in the four municipalities, were invited by a letter to the legal guardian to participate in the study. In Sweden, children with disabilities are generally integrated in mainstream, state-run, schools, either in class or by having special schools located in the same building as the main school. A passive consent model was used for recruitment, where legal guardians could opt their child out of the study. Analysis of school records showed that the total recruited sample, comprising both panels, did not significantly differ from the total population in terms of sex, school absence, or school grades.

The current study draws on just one of the two panels, as the panel in question had been canvassed using the above measures at ages 13, 15, and 17 whereas the other panel had not. Further, participants included in the current study also had to have at least two completed questionnaires for the three waves of data collection. This resulted in a final sample of *n* = 734. The proportion of males and females at t1 was approximately even. The majority (80.7%) of the sample were born in Sweden. At t2, more boys than girls dropped out, but the difference was just beyond the level of significance (χ2 = 3.19, df = 1, *p* = 0.07). At t3, more boys than girls also dropped out compared with the sample at t1 (χ2 = 36.3, df = 1, *p* < 0.01). Participants with a D/CC did not drop out more so than participants without a D/CC at either t2 or t3 (t2: χ2 = 0.01, df = 1, *p* = 0.9; t3: χ2 = 1.38, df = 1, *p* = 0.24). There were also no statistically significant differences in family affluence at t1 between participants and non-participants at either t2 or t3 (χ2 = 3.33, df = 2, *p* = 0.19; χ2 = 3.29, df = 2, *p* = 0.19).

Internal drop-out was thus presumed to be missing-at-random (MAR) for the main measures under study, except for sex. Previous studies have shown that girls tend to have higher rates of emotional symptoms than boys (Inchley et al., 2020). Given the potential interaction effects of higher retention rates of girls at t3 (late adolescence) on the measurement of emotional symptoms, a two-way ANOVA was run with sex and participation at t3 as factors and emotional symptoms as the dependent variable. While sex was significant in the model (F = 46.4; *p* < 0.01), there was no interaction effect with participation at t3 (F = 2.6; *p* = 0.1). This suggests that the measurement of emotional symptoms should not be overtly affected by the higher retention rate for girls.

### Preliminary Data Analyses

Descriptive statistics were produced for the main measures and for each time point (see Table 1). Differences in the peer victimization (non-parametric) and emotional symptoms (parametric) measures between the D/CC and non-D/CC group were tested for each time point (see Table 2). Bivariate correlations were run between peer victimization and emotional symptoms (see Table 3) and the intraclass coefficients (ICCs) were calculated. A threshold for the ICC of >0.1 was set to determine the need for partitioning within- and between-person variance, as simulation studies have found that even with an ICC of 0.1, type 1 errors can be higher than 5% and thus multilevel analysis is therefore warranted (Musca et al., 2011).

**Table 1:** Descriptive statistics for the sample over three time points

Timepoint	*n* (% final sample)	Boys	Girls	D/CC (*n*/%)		Family affluence (*n*/%)	
		*n*/%	*n*/%	Yes	No		
t1 (age 13)	734	355/48.4	379/51.6	225/30.7	509/69.3	More money …	149/20.3
						The same ..	472/64.3
						Less money	98/13.4
t2 (age 15)	533 (72.6%)	247/46.3	286/53.7	164/30.8	369/69.2	More money …	100/19.0
						The same ..	352/67.0
						Less money	73/13.9
t3 (age 17)	386 (52.6%)	146/37.8	240/62.2	111/28.8	275/71.2	More money …	76/20.1
						The same ..	258/68.3
						Less money	44/11.6

**Table 2:** Descriptive statistics for peer victimization and emotional symptoms by D/CC at t1, t2, and t3

Peer victimization				Emotional symptoms			
	D/CC	No D/CC	*U*	D/CC	No D/CC	*t*	df
t1 (age 13)							
Mean	1.34	1.19	43,140**	1.79	1.50*	7.9**	723
95% C.I.	1.29–1.39	1.17–1.22		1.69–1.89	1.45–1.55		
S.D.	0.39	0.28		0.49	0.39		
Median	1.25	1.00		1.80	1.40		
t2 (age 15)							
Mean	1.18	1.11	25,088**	1.84	1.61	4.9**	531
95% C.I.	1.13–1.23	1.09–1.13		1.72–1.96	1.56–1.67		
S.D.	0.31	0.19		0.56	0.42		
Median	1.00	1.00		1.60	1.60		
t3 (age 17)							
Mean	1.10	1.07	14,004 (n.s.)	1.90	1.70	3.2**	384
95% C.I.	1.07–1.14	1.05–1.09		1.8–2.02	1.63–1.77		
S.D.	0.19	0.15		0.53	0.50		
Median	1.00	1.00		1.80	1.60		

*U* Mann–Whitney *U*, *T* Student’s *t* test

*p < 0.05; **p < 0.01

**Table 3:** Correlations between peer victimization and emotional symptoms at three time points

	1	2	3	4	5	6
1. t1 peer victimization	–					
2. t2 peer victimization	0.42**	–				
3. t3 peer victimization	0.14*	0.14*	–			
4. t1 emotional symptoms	0.38**	0.22**	0.17**	–		
5. t2 emotional symptoms	0.26**	0.34**	0.11	0.53**	–	
6. t3 emotional symptoms	0.16**	0.15**	0.20**	0.43**	0.63**	–

*p < 0.05; **p < 0.01

### Main Data Analyses

Random-intercept cross-lagged panel models (RI-CLPM) with a time-invariant predictor (see Mulder & Hamaker, 2021) were used. Contrary to a traditional cross-lagged panel model, the RI-CLPM separates within- from between-person variance, allowing for analysis of temporal, developmental processes separate from the influence of trait-like factors, which can be deemed stable during the study period (see Hamaker et al., 2015). Studying the strength of within-person relations helps understand the effect of temporal processes between peer victimization and emotional symptoms *in relation to individuals’ own developmental trajectories* (see Curran & Bauer, 2011; Hamaker et al., 2015), i.e. the strength of the link between peer victimization and emotional symptoms over and above pre-existing levels of emotional symptoms or peer victimization. The RI-CLPM thus estimates the within-person cross-lagged relations between peer victimization and emotional symptoms at the at the three different time points studied.

A series of models were then run. For the first research question, an RI-CLPM of the mean raw scores of peer victimization and emotional symptoms was estimated, with measurement invariance tests by sex and family affluence. For the second research question, a measurement invariance test was run by D/CC. This is to determine whether both between- and within-person parameters were invariant for adolescents with and without a D/CC. An RI-CLPM was also estimated with D/CC as an external predictor of the observations over all three waves. This estimated the developmental relations between a D/CC and peer victimization or emotional problems over the period 13–17 years old. The model was estimated firstly with D/CC fixed to equality over time, and then freely estimated at each time point. Measurement invariance tests of moderators of the final model were run based on the results to the first research question.

Missing answers were handled using full information maximum likelihood, which allows estimation of missing data if presumed MAR. Because of non-normality in the peer victimization measure, robust estimators (Huber-White) were used, which provide reliable estimates even with non-normal data (see Finney & DiStefano, 2006; Rhemtulla et al., 2012). Following Little (2013), absolute model fit was assessed using robust chi-square (χ2), and comparative fit was assessed using: robust Comparative Fix Index (CFI), Tucker-Lewis Index (TLI), loglikelihood (LL), AIC, BIC, Root Mean Square Error of Approximation (RMSEA), and Standardized Root Mean Square Residual (SRMR). Comparison of nested models (for invariance testing) was assessed using chi² difference (Δχ2) alongside a change of >0.01 in robust CFI (ΔCFI), as recommended by Cheung and Rensvold (2002). This is because using Δχ2 alone has been associated with finding higher levels of invariance (Putnick & Bornstein, 2016). Modeling was conducted in RStudio (2021.09.1 Build 372) using lavaan 0.6-7 (see Rosseel, 2012) and used code provided by Mulder and Hamaker (2021).

## Results

### Preliminary Analyses

Table 1 provides descriptive statistics for the final sample for all three waves (t1, t2, t3).

Table 2 shows descriptive statistics for the main measures of peer victimization and emotional symptoms, by D/CC group, at the three time points. Levels of peer victimization at t1 did not differ between participants and non-participants at t2 (Mann-Whitney U = 49833, *p* = 0.39). At t3, however, participants had slightly lower t1-levels of peer victimization (mean rank 349) compared to those who had dropped out (mean rank 376) (Mann-Whitney U = 60433, *p* = 0.07). In terms of emotional symptoms at t1, there were no differences of note between participants and those who dropped out at either t2 or t3 (t = 0.33, df = 574, *p* = 0.75; t = 0.36, df = 723, *p* = 0.72).

Table 3 shows that correlations between peer victimization and emotional symptoms were significant for all stability and cross-lagged relations at each time point.

Due to skew on the peer victimization measure, non-parametric correlations (Kendall’s tau-b) were also run and showed a similar pattern (see Supplementary Table 1). The ICC for emotional symptoms was 0.5, suggesting that 50% of the variance over time could be accounted for by developmentally stable (during the study period) between-person differences. The ICC for peer victimization was lower at 0.26, implying that 26% of the variance over the measurement points was due to between-person factors. This suggests individual differences play less of a role in the developmental trajectories of peer victimization. The ICC for both measures (being >0.1) justified analysis using a random-intercept model to partition the between- and within-person variance.

### Main Analyses

#### RI-CLPM of the co-development of peer victimization and emotional symptoms

Model fit for the first RI-CLPM of peer victimization and emotional symptoms was good (robust χ2 = 1.11, df = 1, *p* = 0.29; robust CFI = 1.00; TLI = 0.99; LL = −829.15; AIC = 1710.3; BIC = 1829.8; RMSEA = 0.01; SRMSR = 0.01). One non-significant variance was found in the random-intercept for peer victimization (*p* = 0.27), but this was judged to be an effect of skew in the measure. The regression and correlation model parameters are shown in Table 4.

**Table 4:** Parameters for RI-CLPM of peer victimization and emotional symptoms

	*B*	S.E.	*z*	*p*	*β*
Regressions					
t1 PV–t2 PV	0.24	0.09	2.86	<0.01	0.35**
t2 PV–t3 PV	−0.03	0.16	−0.19	0.85	−0.05
t1 ES–t2 ES	0.25	0.19	1.32	0.19	0.22
t2 ES–t3 ES	0.54	0.09	6.20	<0.01	0.49**
t1 PV–t2 ES	0.16	0.10	1.57	0.12	0.13
t2 PV–t3 ES	−0.16	0.15	−1.01	0.31	−0.07
t1 ES–t2 PV	−0.01	0.5	−0.13	0.90	−0.01
t2 ES–t3 PV	−0.01	0.04	−0.36	0.72	−0.04
Correlations					
t1 PV–t1 ES	0.04	0.01	4.65	<0.01	0.38**
t2 PV–t2 ES	0.02	0.01	3.13	<0.01	0.24**
t3 PV–t3 ES	0.01	0.01	1.19	0.23	0.10
RI PV–RI ES	0.01	0.01	2.28	0.02	0.54*

*RI* random intercept

*p < 0.05; **p < 0.01

The random intercepts for peer victimization and emotional symptoms were strongly correlated (r = 0.54). This suggests that between-person factors are an important part of the co-development of peer victimization and emotional symptoms for general adolescents. At a within-person level, contemporaneous correlations were weaker: at age 13 (t1) r = 0.38 and at age 15 (t2) r = 0.24. By age 17 (t3), the correlation (between the residuals) was non-significant. Cross-lagged relations over the two-yearly measurement points were not found at the within-person level. This suggests that if peer victimization can lead to emotional symptoms (or vice versa), then such relations might occur within shorter time periods than two years. Stability (autoregressive) relations were significant at the within-person level for peer victimization between age 13 and 15 (0.35), and for emotional symptoms between age 15 and 17 (0.49). In other words, adolescents who were victimized at age 13 had a slightly higher probability of being victimized two years later, in relation to their own initial levels of victimization. Adolescents with emotional symptoms at age 15 had a higher probability of having emotional symptoms two years later, in relation to their own developmental trajectory.

#### Testing moderators of the model: sex and family affluence

Measurement invariance tests by sex showed that a model with parameters constrained to be equal across sex had a poorer fit (robust χ2 = 35.52, df = 10, *p* < 0.01; robust CFI = 0.95), compared to a model where parameters were freely estimated by sex (robust χ2² = 3.99, df = 2, *p* = 0.14; robust CFI = 1.00; Δχ2 = 31.23, Δdf = 2, *p* < 0.01; ΔCFI = 0.05). Groupwise models run by sex showed that the model for girls retained acceptable fit (robust χ2 = 0.44, df = 2, *p* = 0.8), while the model for boys showed a deterioration in fit, although the absolute fit was still significant (robust χ2 = 3.55, df = 2, *p* = 0.17).

**Fig. 1:**
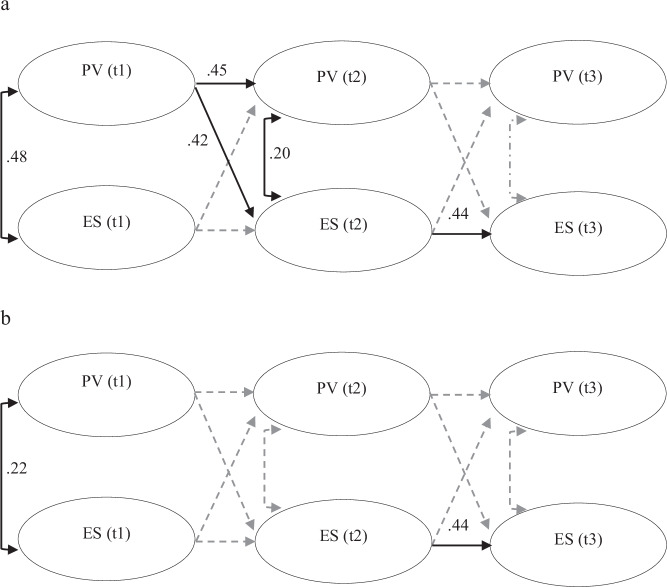
**a** RI-CLP model (within-person standardized parameters) for girls. **b** RI-CLP model (within-person standardized parameters) for boys

Figure 1a, b shows the standardized within-person parameters from the RI-CLPMs for girls and boys. Only significant parameters are shown; non-significant relations are displayed with dashed, grayed-out arrows (see Supplementary Table 2a, b for the full parameter estimates for both models). The RI-CLPM was judged to function less well for boys, due to non-significant variance in both of the random-intercepts, where this was only the case for peer victimization for girls. This implies that boys have more homogeneity (fewer between-person differences) regarding the co-development of peer victimization and emotional symptoms.

For boys, there were no significant within-person cross-lagged relations between peer victimization and emotional symptoms, as measured over the two-year intervals. Contemporaneous correlations existed only at the first time point for both sexes. For girls, there was a different pattern. Firstly, the acceptable fit of the RI-CLPM for girls suggests that there is greater heterogeneity among girls than boys concerning the co-development peer victimization and emotional symptoms. Secondly, a cross-lagged relation was found from peer victimization at age 13 (t1) to emotional symptoms at age 15 (t2), suggesting a temporal ‘spill-over’ effect of peer victimization. Contemporaneous correlations existed only at t1 and t2. Stability relations were found for peer victimization only between t1 and t2, and for emotional symptoms only between t2 and t3.

Measurement invariance tests by family affluence showed that a model with parameters constrained to be equal across the three levels of family affluence had a good fit (robust χ2 = 14.44, df = 19, *p* = 0.76; robust CFI = 1.00), compared to a model where parameters were freely estimated (robust χ2 = 2.45, df = 3, *p* = 0.49; robust CFI = 1.00; Δχ2 = 11.99, df = 16, *p* = 0.75; ΔCFI = 0). Thus, the model was deemed to be invariant at the three different levels of family affluence. This suggests that the co-development of peer victimization and emotional symptoms is not moderated by perceived family affluence.

#### Testing the model by disability/chronic condition

**Fig. 2:**
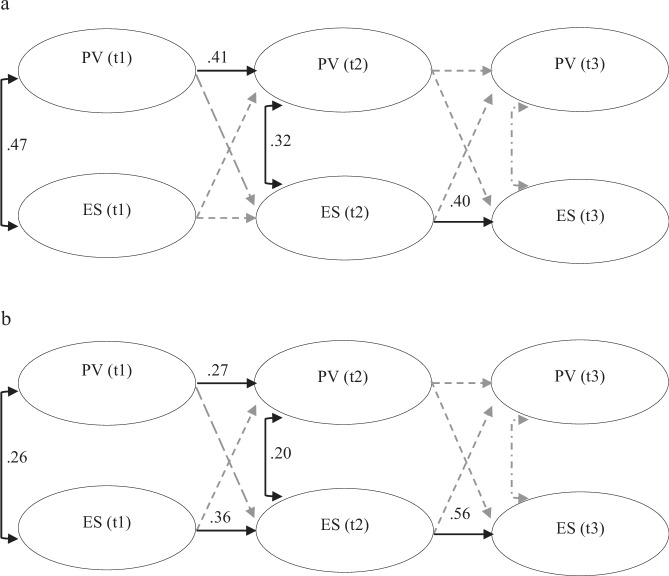
**a** RI-CLP model (within-person standardized parameters) for adolescents with a D/CC. **b** RI-CLP model (within-person standardized parameters) for adolescents without a D/CC

Measurement invariance tests by D/CC showed that a model with parameters constrained to be equal across D/CC groups had a poor fit (robust χ2 = 28.52, df = 10, *p* < 0.01; robust CFI = 0.97), compared to a model where parameters were freely estimated by D/CC (robust χ2 = 0.81, df = 2, *p* = 0.66; robust CFI = 1.00): Δχ2 = 27.71, df = 8, *p* < 0.01; ΔCFI = 0.03). The model was thus judged not invariant by D/CC group. Groupwise models run by D/CC showed that the model for adolescents without a D/CC had marginally better fit (robust χ2 = 0.04, df = 2, *p* = 0.98) than the model for adolescents with a D/CC (robust χ2 = 0.80, df = 2, *p* = 0.67). Figure 2a, b shows the within-person standardized parameters from the RI-CLPM for adolescents with and without a D/CC (see Supplementary Table 3a, b for the full parameter estimates).

For adolescents with a D/CC, there was a strong correlation (0.48, *p* < 0.01) between the random-intercepts, whereas there was not for adolescents without a D/CC. This implies that between-person levels of peer victimization are in general correlated with levels of emotional symptoms for adolescents with a D/CC, but not so for adolescents without a D/CC. The contemporaneous correlations (at t1 and t2) were stronger for adolescents with a D/CC, as was the stability relation between t1 and t2 for peer victimization. This lends support to the idea that adolescents with D/CCs fare ‘worse’ at the temporal (within-person) level concerning the co-development of peer victimization and emotional symptoms. However, adolescents without a D/CC showed stronger stability relations for the development of emotional symptoms across all three time points, suggesting differential dynamic processes for these adolescents over and above their ordinary levels of emotional symptoms.

#### Final model: RI-CLPM with D/CC as an external predictor

**Fig. 3:**
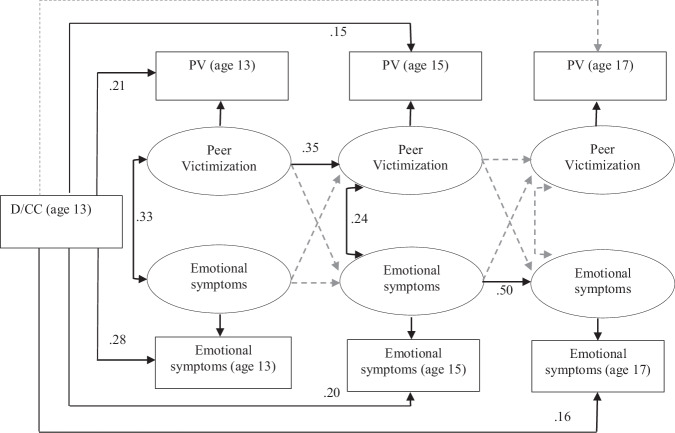
RI-CLPM of the developmental relations between D/CC, peer victimization, and emotional symptoms in adolescence

A final model with D/CC as an external, time-invariant predictor of the observed variables, first fixed to equality and then freely estimated at each time point, showed that the constrained model had a poor fit (robust χ2 = 15.6, df = 5, *p* < 0.01; robust CFI = 0.98; TLI = 0.93; LL = −800.89; AIC = 1657.77; BIC = 1786.53; RMSEA = 0.05; SRMSR = 0.03). The model with D/CC freely varying at each time point provided a better fit (robust χ2 = 0.8, df = 1, *p* = 0.7; robust CFI = 1.00; TLI = 1.01; LL = −792.35; AIC = 1649; BIC = 1796; RMSEA < 0.01; SRMSR = < 0.01; Δχ2 = 14.08, df = 4, *p* < 0.01; ΔCFI = 0.02). This supports the idea of a developmental, i.e. time-varying, relation between D/CC, peer victimization, and emotional symptoms. Figure 3 shows the standardized parameters for the within-person part of the model (see Supplementary Table 4 for the full parameter estimates).

The regression coefficients for relations between D/CC and peer victimization were significant at t1 (age 13) and t2 (age 15), but not at t3 (age 17). It is important to note that these are not causal effects. An interpretation of the coefficients is that having a D/CC increases the likelihood of being victimized at the same age (age 13) by 0.21 standardized units, or by 0.15 units regarding the likelihood of being victimized by age 15 (an increase of 0.21 standardized units represents nearly half a step up on the peer victimization scale, from, for example, never having been victimized to being victimized at least once). A decreasing effect over time of having a D/CC can also be seen; by age 17 having a D/CC did not appear to have a significant relation to being victimized.

A similar decreasing effect was discerned for the relation between D/CC and the development of emotional symptoms. The link between D/CC and emotional symptoms was significant at all three time points. This implies that having a D/CC is linked to an increased likelihood of having emotional symptoms throughout the teenage years, even though the effect lessens towards late adolescence. The relation between D/CC and emotional symptoms was strongest at t1 (age 13), which at 0.28 represents over half a step up on the emotional symptoms scale, e.g., having a D/CC changes the likelihood from not having emotional symptoms halfway towards experiencing them some of the time.

#### Moderation of the final model by sex

As sex, but not family affluence, was found to be a moderator of the first model, measurement invariance tests of the final model were run only by sex. Tests of the final model found that constraining the lagged parameters to be equal across sex had a poorer fit (robust χ2 = 35.70, df = 10, *p* < 0.01; robust CFI = 0.95), compared to a model where parameters were freely estimated by sex (robust χ2 = 3.83, df = 2, *p* = 0.15; robust CFI = 1.00: Δχ2 = 31.87, df = 8, *p* < 0.01; ΔCFI = 0.05). Groupwise models run by sex showed that the model for girls retained acceptable fit (robust χ2 = 0.24, df = 2, *p* = 0.89), while the model for boys showed a deterioration in fit, although the absolute fit was still significant (robust χ2 = 3.59, df = 2, *p* = 0.17). A similar problem to the first model was identified; the variance for both the random-intercepts for boys was non-significant, as was the covariance (see Supplementary Table 5a, b for the full parameter estimates). Again, this suggests that between-person differences concerning the developmental relations between D/CC, peer victimization, and emotional symptoms may be negligible for boys, i.e. there is greater homogeneity among boys.

In general, estimates of D/CC as a predictor of the observed variables did not differ from the model estimates for both sexes combined, apart from on two key points. The link between D/CC and peer victimization at age 15 remained absent for boys but was significant for girls (0.18 standardized estimate). The link between D/CC and emotional symptoms at age 17 disappeared for boys whereas it remained for girls (0.14 standardized estimate).

## Discussion

A link between peer victimization, emotional symptoms, and disability or chronic condition in adolescence is well documented (Bjereld et al., 2022; Hatton et al., 2018), but less is known about the role of stable, individual differences compared to temporal, dynamic processes during adolescent development. Knowledge of the role of individual differences, compared to temporal processes, may help understand the causal mechanisms driving the development of peer victimization and emotional symptoms. To address this gap, the present study examined the developmental relations between peer victimization, emotional symptoms, and disability or chronic condition during adolescence using three waves of self-report panel data from Swedish adolescents at ages 13, 15, and 17. A novel contribution of the study is that estimates are provided, at both between- and within-person levels, of relations between peer victimization and emotional symptoms, and their links to disability or chronic condition throughout adolescence. While within-person estimates are more appropriate for studying individuals’ temporal developmental processes, between-person estimates are essential for understanding the role of developmentally stable factors. Generally, girls and adolescents with a disability or chronic condition showed more heterogeneity in the co-development of peer victimization and emotional symptoms, with girls being more prone than boys to developing emotional symptoms following peer victimization, and particularly in early adolescence. Girls and adolescents with a disability or chronic condition had elevated within-person levels of both peer victimization and emotional symptoms throughout adolescence.

The present study had two main research questions. The first concerned adolescents in general, and for them developmentally stable factors played an important role in the co-development of peer victimization and emotional symptoms. Regarding the development of emotional symptoms, stable individual differences account for approximately ‘half the story’. This suggests that greater attention to heterogeneity – that adolescents can be quite different from another – is particularly important regarding the development of emotional symptoms. For peer victimization, between-person factors play a lesser role, meaning that temporal processes may better explain the development of peer victimization than individual differences. Both sex and family affluence have previously been found to have a moderating role on peer victimization and emotional symptoms (Kavanagh et al., 2017; Smith et al., 2019), but the current study found that only sex was an important between-person factor. In general, girls showed more heterogeneity in the co-development of peer victimization and emotional symptoms than boys, and were more prone than boys to the temporal negative effects of being victimized, particularly in early adolescence.

These findings also contribute to the literature by offering a within-person account of the temporal, developmental relations between peer victimization and emotional symptoms during adolescence. Within-person estimates show the strength of relations over and above individuals’ expected developmental trajectories, as well as avoid conflating individual differences with temporal developmental processes (Hamaker et al., 2015). Previous research (e.g., Christina et al., 2021) supports a bi-directional relation between peer victimization and emotional symptoms in adolescents. The findings partly confirm such research; within-person estimates were found that suggest contemporaneous links at age 13 and age 15. The results, however, extend previous research by suggesting that while no within-person cross-lagged relations were found when grouping both boys and girls together, girls victimized at age 13 had higher levels of emotional symptoms at age 15. This suggests that girls in early adolescence who experience peer victimization may be more likely to develop emotional symptoms that persist two years later over and above pre-existing levels of emotional symptoms. Further, while a contemporaneous link between peer victimization and emotional symptoms disappeared by mid-adolescence for boys, it remained for girls. In international studies, peer victimization tends to peak in early adolescence (Kennedy, 2021) and drops during the later high school period. In Sweden, statutory school ends at age 15 and most adolescents will then start at a new upper secondary/high school (*gymnasium*). This transition provides a natural shift in classroom and school composition. This transition occurred between timepoints two and three in the data, which could also be part of the explanation for changes in levels of peer victimization, i.e. victims and perpetrators from the same school-class or school may have ended up at different high schools.

Stability relations at the within-person level – what Mulder & Hamaker (2021) call within-person carry-over effects – concerning peer victimization were only present in early adolescence (age 13–15). Concerning emotional symptoms, they were only present in later adolescence (age 15–17). These findings generally align with the view that peer victimization is predominantly a phenomenon of early adolescence (Biswas et al., 2020; Kennedy, 2021), while emotional symptoms tend to increase in late adolescence (Inchley et al., 2020). The findings, however, show that these patterns also are present at the level of temporal, within-person development, i.e. after accounting for stable, individual differences. This has ramifications for the question of *when* in development state versus trait plays a role. For example, the state-wise development of emotional symptoms was present between mid- and late adolescence (with a carry-over estimate of 0.49), suggesting that trait-like factors play a stronger role in early adolescence. Sex differences in stability relations were also found, with only girls showing stability (0.45) in peer victimization between early and mid-adolescence. This suggests that victimized girls have an increased probability of *continuing* to be victimized in mid-adolescence, alongside raised levels of emotional symptoms in mid- and late adolescence.

The two-year measurement interval may explain the lack of within-person cross-lagged relations generally – and for boys specifically. It is possible that being victimized led to increased emotional symptoms in the short term, but that such symptoms abated within two years. This presupposes that the period of victimization was also short-lived. However, medium to strong stability relations were found for peer victimization between ages 13 and 15 (for girls and boys), implying that victimization tends to continue, at least for some adolescents, even two years later. This is in line with previous research by, for example Zych et al. (2020). This suggests that the two-year measurement interval should have been able to capture cross-lagged relations had they existed, at least at a general level. What is perhaps being missed is cross-lagged relations for sub-groups of adolescents. Peer victimization may have ceased by age 15 for some adolescents – and thus any effects on emotional symptoms may also have abated – but for other adolescents, victimization continues for most of high school. Investigating sub-groups who are more chronically victimized may be an important area of future investigation, along with studies that use shorter time lags to assess short-term effects of victimization on emotional symptoms. Boys may also respond to victimization with other behaviors, such as externalizing symptoms.

For the second research question, developmentally stable individual differences (between-person factors) played a greater role for adolescents with a disability or chronic condition than for those without, regarding the link between peer victimization and emotional symptoms. Indeed, the lack of a correlation between the random intercepts for adolescents without a disability or chronic condition suggests that, once disability or chronic condition is taken into account, individual differences are a negligible part of the explanation of the co-development in general adolescents of peer victimization and emotional symptoms. Conversely, stable individual differences are likely to be an important part of the explanation concerning adolescents with a disability or chronic condition. Theoretical explanation of the co-development of peer victimization and emotional symptoms may need to pay closer attention to the role of disability and chronic conditions, but also their heterogenous nature.

The results also extend previous research by showing that while peer victimization decreases and emotional symptoms increase for all adolescents, those with a disability or chronic condition have more negative outcomes throughout adolescence, in terms of elevated levels of both peer victimization and emotional symptoms. Moreover, developmental (within-person) relations between these behaviors during early and mid-adolescence were stronger, compared to adolescents without a disability or chronic condition. The link between having a disability or chronic condition and both peer victimization and emotional symptoms was also strongest at age 13. The link was still present, although weaker, at age 15, which was in contrast to the hypothesis (that once between-person factors were controlled for, the links between disability or chronic condition and peer victimization and emotional symptoms would be similar throughout adolescence due to a presumed stability of the effect of having a disability or chronic condition). However, when analyzed by sex, the link between disability or chronic condition and peer victimization at age 15 was absent for boys but significant for girls (0.18 standardized estimate). Further, the link between disability or chronic condition and emotional symptoms at age 17 was not present for boys but was for girls (0.14 standardized estimate). These findings draw attention to the need to consider the intersection of sex and disability or chronic condition in understanding the co-development of peer victimization and emotional symptoms during adolescence.

For boys and girls, the link between disability or chronic condition and peer victimization had disappeared by age 17. An explanation for this is that peer victimization generally tends to become less common during late adolescence, possibly due to victimizers maturing out of their behavior (Álvarez-García et al., 2015; Kennedy, 2021). It may also be the case that victims have had either time or maturational distance from the episode of victimization to overcome psychological difficulties. While these results are thus in line with other studies (e.g., Biswas et al., 2020) that highlight early to mid-adolescence as the key developmental phase in which preventative measures against peer victimization need to be delivered, the findings draw attention to the need for extra or tailored measures for adolescents with a disability or chronic condition who have more negative outcomes concerning the co-development of peer victimization and emotional symptoms.

The present study is also concerned with extending the theoretical, causal account of the co-development of these behaviors, drawing on the empirical analyses. At first sight, peer victimization and emotional symptoms appear to be two different developmental processes, the former starting in early adolescence and decreasing, largely due to temporal factors; the latter increasing throughout adolescence, but due to stable, individual factors in early adolescence equally as much as temporal events in late adolescence. On the one hand, the lack of significant cross-lagged parameters might suggest minimal causal relations within a two-year period, except for girls in early adolescence, even though a number of studies support the link between peer victimization and poorer mental health outcomes (e.g. Gini & Pozzoli, 2009; Modin et al., 2015). On the other hand, strong within-time correlations – which were stronger for girls and adolescents with a disability or chronic condition – suggest that there may be some causal mechanism at work where being victimized leads to increased emotional symptoms, particularly for girls and adolescents with a disability or chronic condition. An explanation might lie in a ‘cumulative adversity mechanism’ (see Hatton et al., 2018), in that girls who may be exhibiting pre-existing emotional symptoms, or adolescents with a disability or chronic condition, are more likely to become targets for peer victimizers. The cumulative effect of peer victimization on already existing difficulties may have an amplification effect on emotional symptoms. Further work may wish to explore these suppositions.

Drawing on a resilience resource systems perspective (Ungar, 2011, 2018), part of the explanation may also reside in that ordinary socio-ecological resources that victimized adolescents could access to seek support are not sufficiently set up for or responsive to girls and adolescents with a disability or chronic condition. For example, a common protective effect (against victimization) for adolescents in general, but also specifically for those with a disability or chronic condition, is having a positive relationship with teachers (Blum et al., 2001; Bjereld et al., 2022). Adolescents with a disability or chronic condition, however, may face additional barriers to accessing and forming such relationships (Hwang et al., 2015; Willis & Granlund, 2020). Future research could further explore whether the quality of potentially protective relationships is different for adolescents with a disability or chronic condition, and whether access to, and quality of, protective relationships moderates the co-development of peer victimization and emotional symptoms. This knowledge could be also useful both in anti-bullying practice, e.g., in schools, and in promoting mental well-being in adolescents, which may need be flexible and differentiated to be sensitive to greater heterogeneity in individual differences, especially for girls. In particular, anti-bullying policy and practice may benefit from specifically addressing measures to support victimized girls in early adolescence, as well as adolescents with a disability or chronic condition generally.

Although the current study presents novel findings, some potential limitations should be acknowledged. The final study sample is presumed to be representative of the wider population from which it was drawn, in that it did not significantly differ from the total population in terms of sex, school absence, or school grades. It was not possible, however, to check for differences between the study sample and the total population concerning the behaviors under study. It was also not possible to check for differences between the two panels or between the final sample and those excluded because of only one completed questionnaire. Such differences are, however, deemed to have minimal impact on the results. Further, although missing data analysis suggested that the data was MAR, except for sex at t3 (age 17) and marginally lower baseline levels of peer victimization in those retained in the study (though this was not statistically significant), other unexamined factors may be affecting the data. The effect of fewer boys participating at t3 was addressed by running separate models by sex. The marginally lower baseline levels of peer victimization in those retained in the study were not statistically significant and deemed of negligible influence on the results.

The results are in the context of Sweden as a high-income country with a developed welfare state (Lindbom, 2001) and low prevalence rates of peer victimization, in comparison to other high-income western countries (Bjereld et al., 2015; Craig et al., 2009; Due & Holstein, 2008; Molcho et al., 2009). Countries with higher prevalence levels of peer victimization, or with different welfare states may find different strengths of estimates in relation to emotional symptoms and/or disability or chronic condition.

Some measurement and analytical issues should also be noted. Researching peer victimization is fraught with measurement issues (Volk et al., 2017) with individual items often having low validity (Bjereld et al., 2020). In the current study, the measure used only four items and although these captured several specific victimizing behaviors, there is a risk of underreporting as the items did not capture all forms of peer victimization. An item was included regarding cyber-victimization to capture online forms of victimization. Thus, some caution should be used when comparing the findings of the current study to other studies that do not have this item as part of the measure.

The self-report nature of the data may imply that the results stem from the adolescent’s subjective viewpoint. While subjective feelings are an important source, future studies could consider using multiple perspectives (see Casper et al., 2015). The measure of family affluence was subjective and relative (to perceptions of other families’ affluence). It may be a tall order to ask 13-year-olds to make such comparisons accurately. While the findings of the current study suggest that the main model was invariant by family affluence, further study with different measures of family income or parental social economic status would be of benefit.

Some studies define disability using diagnoses or create categories of disability by somatic or psychological symptoms. The current study opted not to do this because both somatic and psychological symptoms can be co-occurring, but also because a focus on adolescents in general with disabilities or chronic conditions was desired. Instead, a self-rated measure of functional difficulty experienced as a result of the disability or chronic condition was used. Theoretically, and from a child-centered perspective, perceived levels of difficulty arising from the disability or chronic condition are likely to provide a level of similarity concerning negative attention from peers. It is hoped that this is a valuable contribution to studying adolescents with disability or chronic condition. Further research may need to increase sample size and statistical power to study sub-categories of disability or chronic condition and their relations to the development of multiple behaviors.

The modeling process may have been affected to a degree by the skew in the peer victimization measure. The use of robust estimators goes someway to reducing bias in parameter estimates, but future studies should look at ways of capturing more variance in peer victimization to allow different modeling techniques.

## Conclusion

While the relationship between peer victimization, emotional symptoms, and disability or chronic condition in adolescence is known, the role of stable, individual differences compared to temporal, dynamic processes during adolescent development is underexplored. The present study provides an important contribution to how stable, individual differences, such as sex and disability/chronic condition, play an important role in the co-development of peer victimization and emotional symptoms. Girls and adolescents with a disability or chronic condition show more heterogeneity in the co-development of peer victimization and emotional symptoms, with girls being more prone than boys to developing emotional symptoms following peer victimization, over and above initial levels. Girls and adolescents with a disability or chronic condition have more negative outcomes throughout adolescence, in terms of elevated levels of both peer victimization and emotional symptoms. Temporal, developmental relations between peer victimization, emotional symptoms and having a disability or chronic condition were strongest in early to mid-adolescence. Perceived family affluence did not moderate the relations between peer victimization and emotional symptoms. Theory and practice may need to pay additional attention to the role of sex, disability and their intersection, as well as age, regarding the development of peer victimization and emotional symptoms. Focusing on the difference between stable, individual differences, such as sex and disability, and temporal dynamic processes, furthers the understanding of the development of peer victimization and emotional symptoms during adolescence.

## Supplementary information

Supplementary Information
