# Supplementary material for: Developmental Relations Between Peer Victimization, Emotional Symptoms, and Disability/Chronic Condition in Adolescence: Are Within- or Between-Person Factors Driving Development?
Source: J Youth Adolesc. 2024 Dec 4;54(5):1063–78. doi: 10.1007/s10964-024-02114-3 (PMC12018631; doi:10.1007/s10964-024-02114-3)
Supplement: Supplementary file 1 — Supplementary Information [file 10964_2024_2114_MOESM1_ESM.docx]

**Developmental Relations Between Peer Victimization, Emotional Symptoms, and Disability/Chronic Condition in Adolescence: Are Within- or Between-Person Factors Driving Development?**

**Supplementary Materials**

Supplementary Table 1 – Non-parametric bivariate correlations between peer victimization and emotional symptoms at three time points

|  | | t1 PV | t2 PV | t3 PV | t1 ES | t2 ES | t3 ES |
| --- | --- | --- | --- | --- | --- | --- | --- |
| t2 PV | K | .28^**^ | - |  |  |  |  |
|  | P | <0.01 |  |  |  |  |  |
|  | N | 520 |  |  |  |  |  |
| t3 PV | K | .072 | .20^*^ | - |  |  |  |
|  | P | .01 | .01 |  |  |  |  |
|  | N | 380 | 314 |  |  |  |  |
| t1 ES | K | .27^**^ | .14^**^ | .09^*^ | - |  |  |
|  | P | <0.01 | <0.01 | <0.01 |  |  |  |
|  | N | 718 | 519 | 378 |  |  |  |
| t2 ES | K | .18^**^ | .23^**^ | .12 | .40^**^ | - |  |
|  | P | <0.01 | <0.01 | .06 | <0.01 |  |  |
|  | N | 526 | 525 | 316 | 526 |  |  |
| t3 ES | K | .11^**^ | .09** | .16^**^ | .33^**^ | .49^**^ | - |
|  | P | <0.01 | .01 | <0.01 | <0.01 | <0.01 |  |
|  | N | 384 | 317 | 382 | 382 | 320 |  |
| K = Kendall’s tau-b  P = Two-tailed significance  * p < 0.05, ** p < 0.01 | | | | | | | |
|  | | | | | | | |
|  | | | | | | | |

Supplementary Table 2a – Parameters for RI-CLPM of peer victimization (PV) and emotional symptoms (ES) for girls

| *Regressions* | B | S.E. | z | p | β |
| --- | --- | --- | --- | --- | --- |
| t1 PV - t2 PV | 0.36 | 0.12 | 3.08 | <0.01 | 0.45** |
| t2 PV - t3 PV | -0.02 | 0.07 | -0.30 | 0.75 | -0.03 |
| t1 ES - t2 ES | -0.01 | 0.23 | -0.01 | 0.99 | -0.01 |
| t2 ES - t3 ES | 0.44 | 0.10 | 4.27 | <0.01 | 0.44** |
| t1 PV - t2 ES | 0.51 | 0.15 | 3.46 | <0.01 | 0.42** |
| t2 PV - t3 ES | -0.11 | 0.16 | -0.67 | 0.50 | -0.07 |
| t1 ES - t2 PV | -0.02 | 0.07 | -0.32 | 0.75 | -0.03 |
| t2 ES - t3 PV | -0.02 | 0.05 | -0.43 | 0.67 | -0.06 |
| *Correlations* | | | | | |
| t1 PV - t1 ES | 0.05 | 0.01 | 4.08 | <0.01 | 0.48** |
| t2 PV - t2 ES | 0.02 | 0.01 | 2.18 | 0.03 | 0.20* |
| t3 PV - t3 ES | 0.01 | 0.01 | 0.19 | 0.85 | 0.03 |
| RI PV -RI ES | 0.01 | 0.01 | 1.77 | 0.08 | 0.49 |
| RI = random intercept | | | | | |

| * p < 0.05, ** p < 0.01 |
| --- |
|  |

Supplementary Table 2b - Parameters for RI-CLPM of peer victimization (PV) and emotional symptoms (ES) for boys

| *Regressions* | B | S.E. | z | p | β |
| --- | --- | --- | --- | --- | --- |
| t1 PV - t2 PV | 0.10 | 0.10 | 1.05 | 0.29 | 0.20 |
| t2 PV - t3 PV | 0.03 | 0.34 | 0.09 | 0.93 | 0.04 |
| t1 ES - t2 ES | 0.40 | 0.22 | 1.78 | 0.08 | 0.40 |
| t2 ES - t3 ES | 0.51 | 0.19 | 2.63 | 0.01 | 0.44** |
| t1 PV - t2 ES | -0.20 | 0.12 | -1.63 | 0.10 | -0.15 |
| t2 PV - t3 ES | -0.32 | 0.47 | -0.68 | 0.50 | -0.11 |
| t1 ES - t2 PV | -0.06 | 0.07 | -0.87 | 0.39 | -0.16 |
| t2 ES - t3 PV | -0.02 | 0.09 | -0.25 | 0.80 | -0.06 |
| *Correlations* | | | | | |
| t1 PV - t1 ES | 0.03 | 0.01 | 2.03 | 0.04 | 0.22* |
| t2 PV - t2 ES | 0.06 | 0.01 | 0.68 | 0.50 | 0.12 |
| t3 PV - t3 ES | 0.01 | 0.01 | 1.31 | 0.19 | 0.15 |
| RI PV - RI ES | 0.01 | 0.01 | 1.30 | 0.19 | 1.76 |
| RI = random intercept | | | | | |

* p < 0.05, ** p < 0.01

Supplementary Table 3a - Parameters for RI-CLPM of peer victimization (PV) and emotional symptoms (ES) for adolescents with a D/CC

| *Regressions* | B | S.E. | z | p | β |
| --- | --- | --- | --- | --- | --- |
| t1 PV - t2 PV | 0.30 | 0.09 | 3.51 | <0.01 | 0.41** |
| t2 PV - t3 PV | -0.43 | 0.26 | -1.6 | 0.11 | -1.2 |
| t1 ES - t2 ES | -0.08 | 0.38 | -0.21 | 0.83 | -0.06 |
| t2 ES - t3 ES | 0.39 | 0.14 | 2.8 | <0.01 | 0.40** |
| t1 PV - t2 ES | 0.19 | 0.21 | 0.92 | 0.36 | 0.16 |
| t2 PV - t3 ES | -0.13 | 0.22 | -0.57 | 0.57 | -0.08 |
| t1 ES - t2 PV | -0.07 | 0.09 | -0.77 | 0.44 | -0.09 |
| t2 ES - t3 PV | -0.01 | 0.07 | -0.18 | 0.86 | -0.06 |
| *Correlations* | | | | | |
| t1 PV - t1 ES | 0.06 | 0.01 | 4.57 | <0.01 | 0.47** |
| t2 PV - t2 ES | 0.03 | 0.01 | 2.56 | 0.03 | 0.32* |
| t3 PV - t3 ES | -0.01 | 0.01 | -0.15 | 0.88 | -0.06 |
| RI PV - RI ES | 0.03 | 0.01 | 3.70 | <0.01 | 0.48 |
| RI = random intercept | | | | | |

* p < 0.05, ** p < 0.01

|  |
| --- |

Supplementary Table 3b - Parameters for RI-CLPM of peer victimization (PV) and emotional symptoms (ES) for adolescents without a D/CC

| *Regressions* | B | S.E. | z | p | β |
| --- | --- | --- | --- | --- | --- |
| t1 PV - t2 PV | 0.17 | 0.06 | 2.74 | <0.01 | 0.27** |
| t2 PV - t3 PV | 0.17 | 0.13 | 1.29 | 0.20 | 0.22 |
| t1 ES - t2 ES | 0.41 | 0.19 | 2.15 | 0.03 | 0.36* |
| t2 ES - t3 ES | 0.64 | 0.10 | 6.20 | <0.01 | 0.56** |
| t1 PV - t2 ES | 0.16 | 0.10 | 1.66 | 0.10 | 0.11 |
| t2 PV - t3 ES | -0.21 | 0.17 | -1.23 | 0.22 | -008 |
| t1 ES - t2 PV | 0.02 | 0.03 | 0.21 | 0.83 | 0.02 |
| t2 ES - t3 PV | 0.01 | 0.04 | 0.17 | 0.87 | 0.02 |
| *Correlations* | | | | | |
| t1 PV - t1 ES | 0.02 | 0.01 | 3.34 | <0.01 | 0.26** |
| t2 PV - t2 ES | 0.01 | 0.01 | 2.84 | 0.03 | 0.20* |
| t3 PV - t3 ES | 0.01 | 0.01 | 1.94 | 0.05 | 0.15 |
| RI PV - RI ES | 0.01 | 0.01 | 0.73 | 0.47 | 0.55 |
| RI = random intercept | | | | | |

* p < 0.05, ** p < 0.01

Supplementary Table 4 - Parameters for RI-CLPM of peer victimization (PV) and emotional symptoms (ES), with D/CC as a time-varying predictor loading on the indicators (i)

| *Regressions* | B | S.E. | z | p | β |
| --- | --- | --- | --- | --- | --- |
| t1 PV -t2 PV | 0.30 | 0.09 | 3.51 | <0.01 | 0.41** |
| t2 PV - t3 PV | -0.43 | 0.26 | -1.6 | 0.11 | -1.2 |
| t1 ES - t2 ES | -0.08 | 0.38 | -0.21 | 0.83 | -0.06 |
| t2 ES - t3 ES | 0.39 | 0.14 | 2.8 | <0.01 | 0.40** |
| t1 PV - t2 ES | 0.19 | 0.21 | 0.92 | 0.36 | 0.16 |
| t2 PV - t3 ES | -0.13 | 0.22 | -0.57 | 0.57 | -0.08 |
| t1 ES - t2 PV | -0.07 | 0.09 | -0.77 | 0.44 | -0.09 |
| t2 ES - t3 PV | -0.01 | 0.07 | -0.18 | 0.86 | -0.06 |
| D/CC - t1 PVi | 0.15 | 0.03 | 5.07 | <0.01 | 0.21** |
| D/CC - t2 PVi | 0.08 | 0.03 | 2.98 | <0.01 | 0.15** |
| D/CC - t3 PVi | 0.03 | 0.02 | 1.44 | 0.15 | 0.08 |
| D/CC - t1 ESi | 0.27 | 0.04 | 7.38 | <0.01 | 0.28** |
| D/CC - t2 ESi | 0.22 | 0.05 | 4.61 | <0.01 | 0.20** |
| D/CC - t3 ESi | 0.18 | 0.05 | 3.32 | <0.01 | 0.16** |
| *Correlations* | | | | | |
| t1 PV - t1 ES | 0.06 | 0.01 | 4.57 | <0.01 | 0.47** |
| t2 PV - t2 ES | 0.03 | 0.01 | 2.56 | 0.03 | 0.32* |
| t3 PV - t3 ES | -0.01 | 0.01 | -0.15 | 0.88 | -0.06 |
| RI PV - RI ES | 0.01 | 0.01 | 2.25 | 0.02 | 0.53* |
| RI = random intercept | | | | | |

* p < 0.05, ** p < 0.01

Supplementary Table 5a - Parameters for RI-CLPM of peer victimization (PV) and emotional symptoms (ES), with D/CC as a time-varying predictor loading on the indicators (i) for girls

| *Regressions* | B | S.E. | z | p | β |
| --- | --- | --- | --- | --- | --- |
| t1 PV -t2 PV | 0.36 | 0.11 | 3.20 | <0.01 | 0.45** |
| t2 PV - t3 PV | -0.03 | 0.16 | -0.17 | 0.86 | -0.05 |
| t1 ES - t2 ES | 0.01 | 0.23 | 0.01 | 0.99 | 0.01 |
| t2 ES - t3 ES | 0.45 | 0.10 | 4.14 | <0.01 | 0.44** |
| t1 PV - t2 ES | 0.48 | 0.14 | 3.45 | <0.01 | 0.40** |
| t2 PV - t3 ES | -0.11 | 0.15 | -0.75 | 0.46 | -0.07 |
| t1 ES - t2 PV | -0.04 | 0.07 | -0.48 | 0.63 | -0.05 |
| t2 ES - t3 PV | -0.02 | 0.05 | -0.39 | 0.70 | -0.05 |
| D/CC - t1 PVi | 0.16 | 0.04 | 4.04 | <0.01 | 0.23** |
| D/CC - t2 PVi | 0.10 | 0.04 | 2.75 | 0.01 | 0.18** |
| D/CC - t3 PVi | 0.03 | 0.02 | 1.23 | 0.22 | 0.09 |
| D/CC - t1 ESi | 0.27 | 0.05 | 5.06 | <0.01 | 0.27** |
| D/CC - t2 ESi | 0.19 | 0.06 | 3.08 | <0.01 | 0.18** |
| D/CC - t3 ESi | 0.15 | 0.06 | 2.34 | 0.02 | 0.14* |
| *Correlations* | | | | | |
| t1 PV - t1 ES | 0.04 | 0.01 | 3.96 | <0.01 | 0.44** |
| t2 PV - t2 ES | 0.02 | 0.01 | 2.19 | 0.03 | 0.20* |
| t3 PV - t3 ES | 0.01 | 0.01 | 0.44 | 0.66 | 0.05 |
| RI PV - RI ES | 0.01 | 0.01 | 1.72 | 0.09 | 0.48 |
| RI = random intercept | | | | | |

| * p < 0.05, ** p < 0.01 |
| --- |
|  |

Supplementary Table 5b - Parameters for RI-CLPM of peer victimization (PV) and emotional symptoms (ES), with D/CC as a time-varying predictor loading on the indicators (i) for boys

| *Regressions* | B | S.E. | z | p | β |
| --- | --- | --- | --- | --- | --- |
| t1 PV -t2 PV | 0.11 | 0.10 | 1.09 | 0.28 | 0.20 |
| t2 PV - t3 PV | 0.07 | 0.32 | 0.23 | 0.82 | 0.08 |
| t1 ES - t2 ES | 0.34 | 0.23 | 1.52 | 0.13 | 0.34 |
| t2 ES - t3 ES | 0.45 | 0.10 | 4.41 | <0.01 | 0.44** |
| t1 PV - t2 ES | -0.21 | 0.12 | -1.70 | 0.09 | -0.16 |
| t2 PV - t3 ES | -0.11 | 0.15 | -0.75 | 0.46 | -0.07 |
| t1 ES - t2 PV | -0.07 | 0.08 | -0.93 | 0.35 | -0.16 |
| t2 ES - t3 PV | -0.02 | 0.05 | -0.39 | 0.70 | -0.05 |
| D/CC - t1 PVi | 0.13 | 0.04 | 3.08 | <0.01 | 0.18** |
| D/CC - t2 PVi | 0.03 | 0.03 | 1.08 | 0.28 | 0.07 |
| D/CC - t3 PVi | 0.03 | 0.03 | 0.78 | 0.44 | 0.07 |
| D/CC - t1 ESi | 0.25 | 0.05 | 5.28 | <0.01 | 0.28** |
| D/CC - t2 ESi | 0.19 | 0.06 | 3.14 | <0.01 | 0.21** |
| D/CC - t3 ESi | 0.13 | 0.10 | 1.30 | 0.19 | 0.13 |
| *Correlations* | | | | | |
| t1 PV - t1 ES | 0.04 | 0.01 | 4.00 | <0.01 | 0.44** |
| t2 PV - t2 ES | 0.15 | 0.01 | 2.19 | 0.03 | 0.20* |
| t3 PV - t3 ES | 0.02 | 0.01 | 0.44 | 0.66 | 0.05 |
| RI PV - RI ES | 0.01 | 0.01 | 1.72 | 0.09 | 0.48 |
| RI = random intercept | | | | | |

* p < 0.05, ** p < 0.01
